# Hydroxytyrosol Alleviates Acute Liver Injury by Inhibiting the TNF-α/PI3K/AKT Signaling Pathway via Targeting TNF-α Signaling

**DOI:** 10.3390/ijms252312844

**Published:** 2024-11-29

**Authors:** Zhining Gao, Haoyang Dai, Qinqin Zhang, Fan Yang, Chenxi Bu, Suiqing Chen

**Affiliations:** 1 College of Pharmacy, Henan University of Chinese Medicine, 156 Jinshui East Road, Zhengzhou 450046, China; 2 Henan Key Laboratory of Chinese Medicine Resources and Chemistry, 156 Jinshui East Road, Zhengzhou 450046, China; 3 Collaborative Innovation Center of Research and Development on the Whole Industry Chain of Yu-Yao, Henan University of Chinese Medicine, Zhengzhou 450046, China

**Keywords:** acute liver injury, hydroxytyrosol, TNF-α, phytotherapy, overexpression

## Abstract

Acute liver injury (ALI) is an injury to liver tissue caused by viruses, drugs, alcohol, and oxygen deprivation, and is one of the most common and serious clinical disorders. Hydroxytyrosol (HT) is a naturally occurring polyphenolic compound isolated from forsythia and has excellent anti-inflammatory properties. However, the effect and mechanisms of HT in ALI remain unclear. We used the LPS/D-GalN induced experimental ALI mouse model and AML12 cells to reveal the efficacy and potential mechanisms of HT in ALI, and HE staining was used for the evaluation of pathologies. A biochemical assay was used to detect changes in liver function, RNA-seq was conducted to reveal the underlying mechanisms of HT for ALI, and WB, RT-qPCR, and IF were used to assess the effects of HT action. Furthermore, an in vitro ALI model against HT in AML12 cells induced by LPS/D-GalN was used to assess the HT protection mechanism. HT significant alleviated LPS/D-GalN-induced ALI in the mice by suppressing inflammatory. In terms of RNA-seq, HT improved the TNF, ECM-receptor interaction, and PI3K/AKT signaling pathway, and it downregulated the mRNA levels of VCAM-1, CXCL5, TNF-α and IL-6 in the liver. Mechanically, HT alleviated LPS/D-GalN in the mice by targeting TNF-α, thereby inhibiting the TNF-α/PI3K/AKT signaling pathway.

## 1. Introduction

As the body’s largest metabolic organ, the liver often faces attacks from various factors, including viruses, trauma, and chemical agents, which are major contributors to different liver diseases [1]. Chemical-induced acute liver injury (ALI) is one of the most common clinical factors that induces severe liver diseases, and it is one of the most common severe diseases seen in medical clinics. ALI pathogenesis includes the loss of the hepatic detoxification function, the massive death of liver parenchymal cells in a short period of time, and uncontrolled inflammation [2], and these processes manifest in the late stage as acute liver failure, which has a high mortality rate and substantial impact on human health [3]. Lipopolysaccharide (LPS) is a common inflammatory stimulus that can cause endotoxin damage, produce inflammatory factors, and lead to hepatocyte apoptosis and necrosis. D-GalN is a sensitizer of LPS that can competitively inhibit intrahepatic uridine nucleotides, inhibit mRNA and protein synthesis, and further exacerbate liver injury [4]. The LPS/D-GalN model has been widely used to induce acute liver injury and facilitate the screening of therapeutic agents targeting this condition. Traditional Chinese medicine is characterized as a multi-component, multi-target, and multi-pathway medical model [5]. Therefore, it is necessary to find the material basis and mechanism of action for the treatment of ALI and to develop effective therapeutic drugs using traditional Chinese medicine.

Hydroxytyrosol (HT) is a natural polyphenolic compound isolated from *Forsythia suspensa* (Thunb.) Vahl with promising pharmacological activities. Initially, the effects of HT were described as the inhibition of Lactobacillus plantarum; however, diverse investigations have revealed its protective role in various diseases in recent years. The combination of HT and paclitaxel enhance antioxidant levels while maintaining the antitumor effectiveness of standard chemotherapy [6]. In bovine mastitis, HT effectively mitigates oxidative stress caused by LPS, thereby reducing the subsequent inflammatory response [7]. Oleuropein-HT-oleocanthal combination therapy has demonstrated potent cardioprotective, antihyperglycemic, and anti-atherosclerotic effects in vivo, offering significant and clinically relevant cardiovascular benefits for high-risk patients [8]. HT alleviates stress-induced liver injury by promoting autophagy through HDAC1/2 inhibition [9]. However, to date, no studies have focused on the potential of HT for the treatment of LPS/D-GalN induced ALI. Based on these grounds, we performed a preliminary assessment of the therapeutic potential of HT to ameliorate ALI.

In this study, we found that HT ameliorated LPS/D-GalN-induced ALI in mice by suppressing inflammation and modulating alterations in serum metabolites. We uncovered that, mechanistically, HT inhibited the LPS/D-GalN-induced activation of the TNF-α/PI3K/AKT signaling pathway. Our findings emphasize the role of HT in directly targeting TNF-α.

## 2. Results

### 2.1. HT Improved Liver Injury in LPS/D-GalN Induced ALI

**Figure 1:**
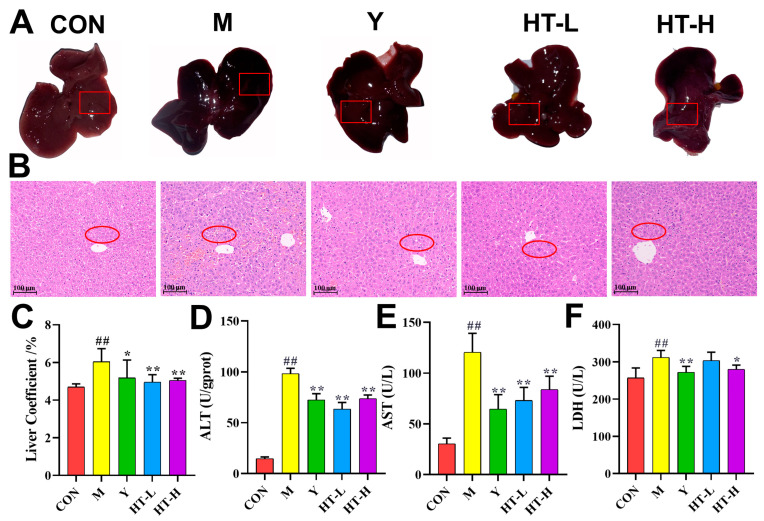
HT ameliorates liver tissue damage in mice with ALI. CON: control; M: LPS&D-GalN; Y: the positive group; HT-L: low-dose hydroxytyrosol; HT-H: high-dose hydroxytyrosol. (**A**) Morphological map of liver tissues (*n* = 6). (**B**) H&E staining of liver tissues (*n* = 6). (**C**) Liver coefficient (*n* = 6). (**D**–**F**) Serum levels of ALT, AST, and LDH in the liver (*n* = 6). Data are expressed as means ± SD; ^##^ *p* < 0.01 compared with the CON group; * *p* < 0.05 and ** *p* < 0.01 compared with the M group.

The liver tissue was red and shiny in the CON group (control group), and liver hemorrhage was more severe in the M group (model group). The M group showed different degrees of improvement in liver hemorrhage and a decrease in the liver coefficient after the administration of HT (Figure 1A,C). As shown in Figure 1B, normal hepatocytes were well aligned and structurally normal, while the M group had inflammatory cell infiltration with wrinkled nuclei, which showed different degrees of ameliorative effects after the administration of HT. When the liver was injured, the transaminases in the hepatocytes entered the blood, and the ALT, AST, and LDH levels in the blood increased. Thus, we tested the ALT, AST, and LDH levels in the blood and found that HT was able to reduce their levels (Figure 1D–F).

### 2.2. HT Regulates the Expression Level of TNF-α Related Proteins in ALI Mice

**Figure 2:**
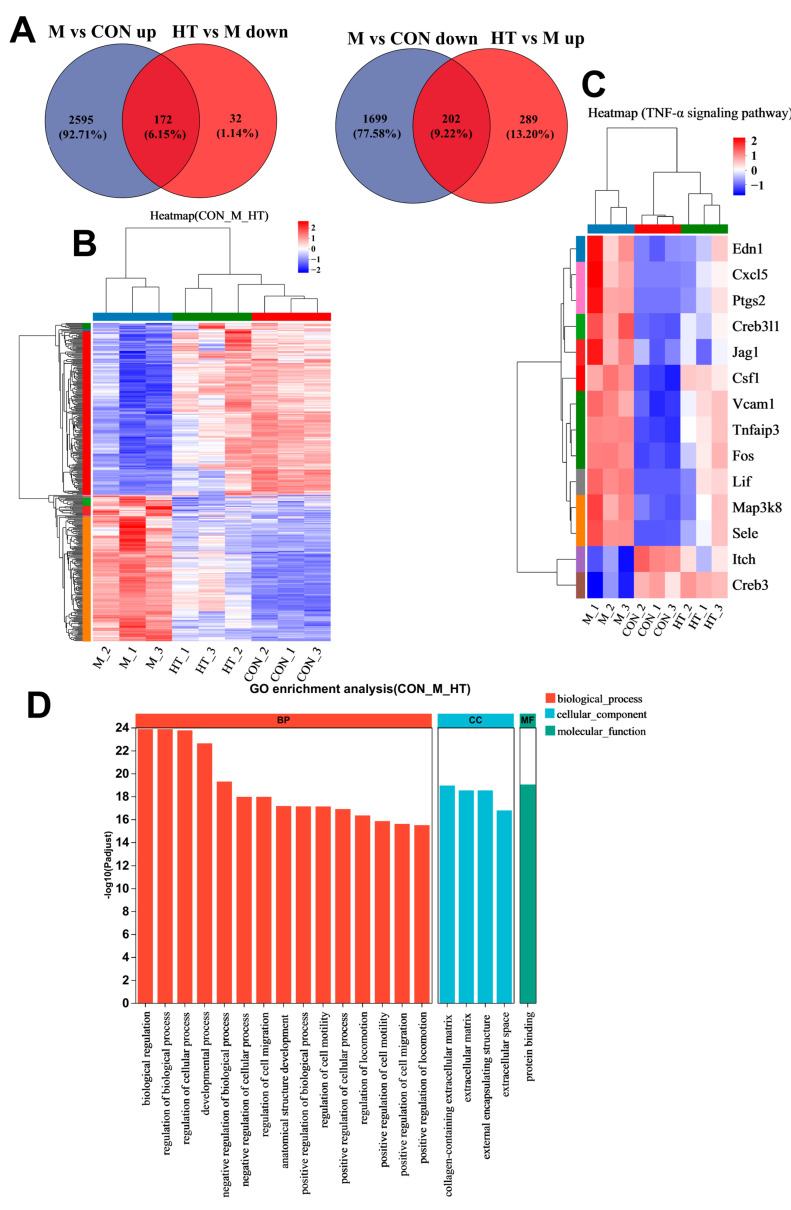
Analysis of differentially expressed genes (DEGs) in ALI. CON: control; M: LPS/D-GalN; HT: high-dose hydroxytyrosol. (**A**) Volcano plot of DEGs in M vs. CON and HT vs. M groups (*n* = 3 per group). (**B**) Heatmap showing the differentially expressed genes from the liver RNA-seq data (*n* = 3). (**C**) Heatmap showing some of the differentially expressed genes of the TNF pathway in the liver RNA-seq data (*n* = 3). (**D**) Results of GO pathway enrichment analysis in CON, M, and HT groups (*n* = 3). Data are expressed as means ± SD.

The M group exhibited significant gene expression changes compared with the CON group, with 1901 downregulated and 2767 upregulated genes. HT treatment reversed this trend, restoring 204 downregulated and 491 upregulated genes. A total of 695 genes were significantly altered in the M group and were also significantly regulated by HT (Figure 2A). The heatmap of genes that were altered in the M group mice and that could be reversed by HT are shown in Figure 2B, and one of the heatmaps for genes in the TNF pathway is shown in Figure 2C. The screened differential genes were subjected to GO function enrichment analysis and were mainly enriched in the following biological processes: (1) cellular component (CC), including collagen-containing extracellular matrix, extracellular matrix, external encapsulating structure, and others; (2) biological processes (BPs), including cellular processes, biological regulation, and others, and; (3) molecular functions (MFs), including protein binding (Figure 2D).

**Figure 3:**
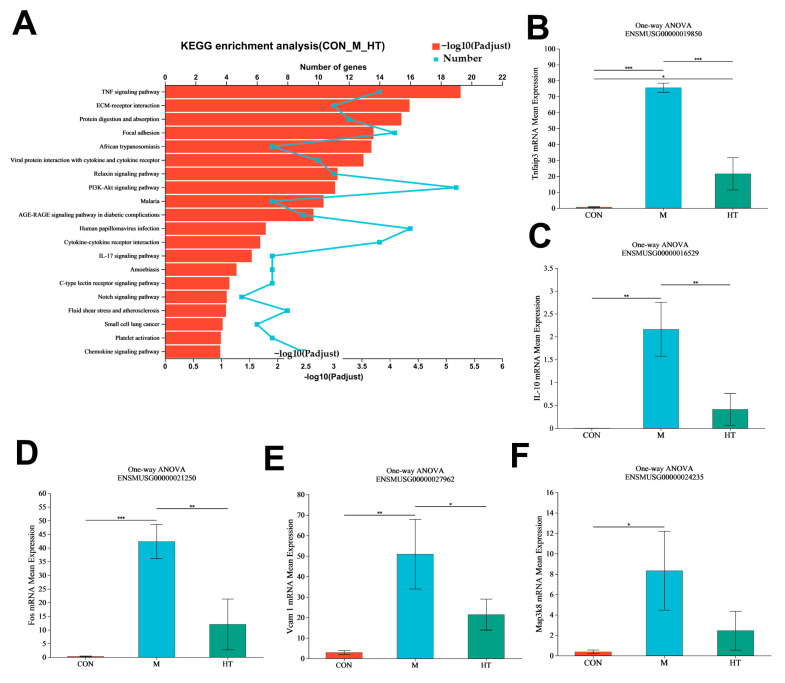
Analysis of differential expression genes (DEGs) in liver tissues of mice in the CON, M and HT group mice. CON: control, M: LPS/D-GalN, Y: the positive group, HT-L: low-dose hydroxytyrosol, HT-H: high-dose hydroxytyrosol. (**A**) KEGG enrichment analysis of DEGs downregulated and upregulated by M but upregulated and downregulated by HT (*n* = 3). (**B**–**F**) The Tnfaip3, IL-10, Fos, Vcam 1, and Map3k8 TPMs in the CON, M and HT group (*n* = 3). Data are expressed as means ± SD; * *p* < 0.05, ** *p* < 0.01 and *** *p* < 0.001 compared with the M group.

These findings suggest that HT’s protective effects against ALI-induced liver injury may be mediated by modulating the TNF-α and PI3K-Akt signaling pathway and the ECM-receptor interaction pathway (Figure 3A). Furthermore, the levels of Tnfaip3, IL-10, Fos, Vcam 1, and Map3k8 protein expression were upregulated in the mice with ALI, and HT treatment resulted in a significant reversal of these changes, with the extent of the reversal varying among the different parameters (Figure 3B–F).

### 2.3. HT Improves Inflammation-Related Gene Expression Levels

**Figure 4:**
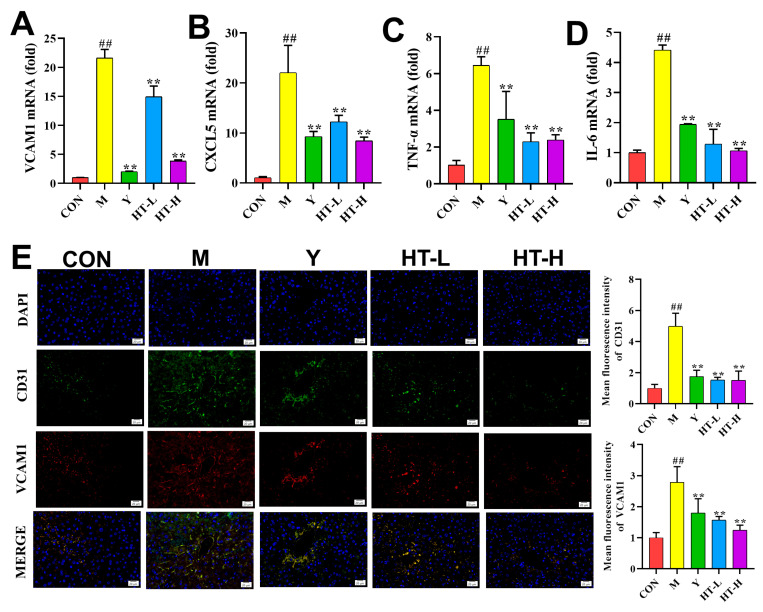
HT ameliorates inflammatory damage in mice with ALI. CON: control; M: LPS&D-GalN; Y: the positive group; HT-L: low-dose hydroxytyrosol; HT-H: high-dose hydroxytyrosol. (**A**–**D**) Effects of HT on the mRNA level of VCAM1, CXCL5, TNF-α and IL-6 (*n* = 6); (**E**) Immunofluorescence analysis of liver tissue sections for CD31 and VCAM1 in mice with ALI (*n* = 6). CON: control; M: LPS/D-GalN; Y: the positive group; HT-L: low-dose hydroxytyrosol; HT-H: high-dose hydroxytyrosol. Data are expressed as means ± SD; ^##^ *p* < 0.01 compared with the CON group; ** *p* < 0.01 compared with the M group.

The qRT-PCR analysis revealed the increased expression of VCAM1, CXCL5, TNF-α, and IL-6 in the M group, which were significantly reversed by HT treatment (Figure 4A–D). The immunofluorescence staining of the liver sections confirmed these findings, demonstrating reduced expression of CD31 and VCAM1 in the HT group compared with the M group (Figure 4E).

### 2.4. HT Regulates TNF-α Signaling Pathway in LPS/D-GalN-Induced ALI

**Figure 5:**
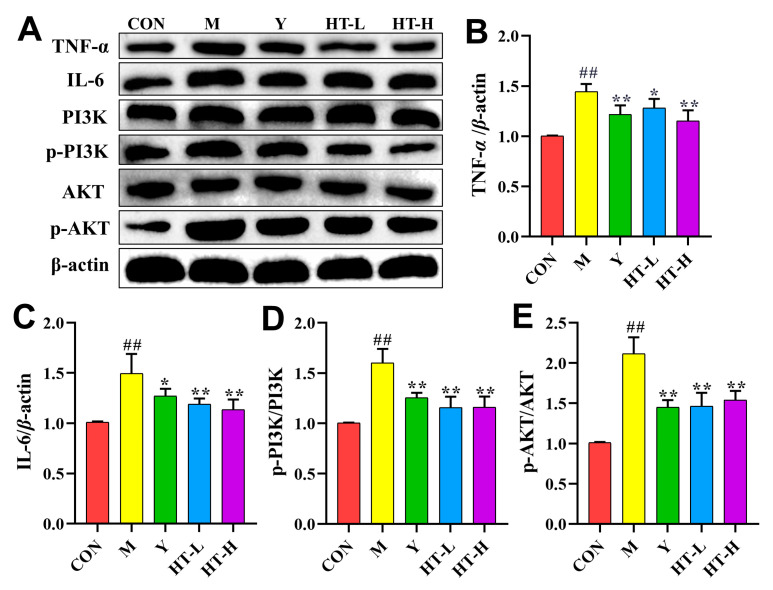
HT regulates the TNF-α signaling pathway in mice with ALI. CON: control; M: LPS/D-GalN; Y: the positive group; HT-L: low-dose hydroxytyrosol; HT-H: high-dose hydroxytyrosol. (**A**) Representative Western blot analysis of TNF-α, IL-6, p-PI3K/PI3K and p-AKT/AKT in the liver (*n* = 3). (**B**–**E**) Protein band intensity of the TNF-α, IL-6, p-PI3K/PI3K and p-AKT/AKT (*n* = 3). CON: control; M: LPS/D-GalN; Y: the positive group; HT-L: low-dose hydroxytyrosol; HT-H: high-dose hydroxytyrosol. Data are expressed as mean ± SD; ^##^ *p* < 0.01 compared with the CON group; * *p* < 0.05 and ** *p* < 0.01 compared with the M group.

In mice with ALI, the levels of TNF-α, IL-6, p-PI3K/PI3K, and p-AKT/AKT were elevated, and HT treatment significantly reversed these increases (Figure 5).

### 2.5. HT Regulates Inflammatory Responses Through the TNF-α Signaling Pathway

**Figure 6:**
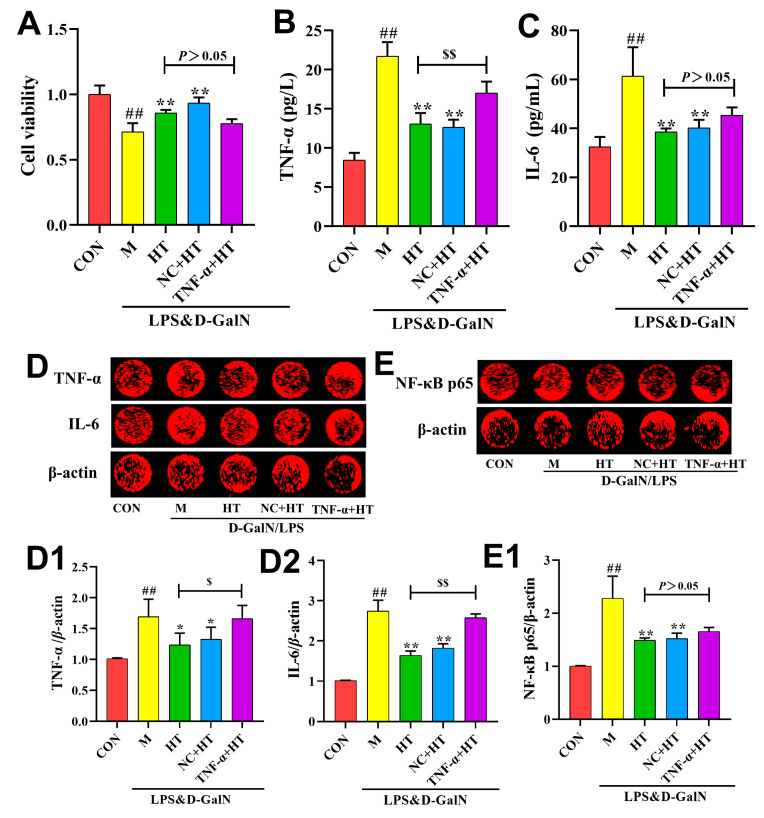
The effect of HT on AML12 cells is associated with TNF-α. (**A**) Cell viability (*n* = 4). (**B**,**C**) The levels of IL-6 and TNF-α Inflammatory factors in AML12 cells (*n* = 6). (**D**,**E**) Relative intensities of TNF-α, IL-6 and NF-κB p65 (*n* = 6). (**D1**,**D2**,**E1**) Protein band intensities of TNF-α, IL-6 and NF-κB p65 (*n* = 6). CON: control; M: LPS/D-GalN; HT: hydroxytyrosol. Data are expressed as means ± SD; ^##^ *p* < 0.01 compared with the CON group; * *p* < 0.05 and ** *p* < 0.01 compared with the M group; ^$^ *p* < 0.05 and ^$$^ *p* < 0.01 compared with the HT group.

As shown in Figure 6, the M group exhibited a significant upregulation of TNF-α, IL-6, and NF-κB p65 protein expression compared with the CON group. The relative expression of TNF-α, IL-6, and NF-κB p65 in the cells of the groups administered HT decreased significantly after the HT intervention, suggesting that it significantly reduced their expression, and there were no significant differences in the relative expression of TNF-α, IL-6, and NF-κB p65 in the liver tissues of the mice in the NC+HT group compared with those in the HT group. TNF-α, IL-6, and NF-κB p65 expression levels were similar in the AML12 cells in the NC+HT and HT groups. The relative expression of TNF-α and IL-6 in the cells of the TNF-α+HT group was significantly higher than that of the HT group, suggesting that TNF overexpression reversed the effect of HT in reducing the expression of TNF-α and IL-6 in AML12 cells. Although the expression level of the NF-κB p65 nuclear protein in the TNF-α+HT group increased compared with that in the HT group, there was no significant difference, suggesting that TNF overexpression could not completely reverse the effect of HT in inhibiting the expression of NF-κB p65 nuclear protein in AML12 cells.

## 3. Discussion

As the largest substantive organ in the body and rich in blood, the liver is susceptible to various irritating and pathogenic factors and stimulants inside and outside the body, leading to the occurrence of liver disease [10]. ALI refers to liver tissue injury caused by viruses, drugs, alcohol, hypoxia, and other factors, and is one of the most common serious clinical diseases [11]. ALI pathogenesis includes the loss of hepatic detoxification function, massive liver parenchymal cell death in a short period of time, uncontrolled inflammation, and the development of the late stage manifests as acute liver failure, with a high mortality rate and a substantial impact on human health [12]. Therefore, research on drugs and methods for preventing and treating liver diseases is of considerable importance. LPS is a common inflammatory stimulus that can cause endotoxin damage and produce inflammatory factors, leading to the apoptosis and necrosis of hepatocytes [13]. D-GalN is an LPS sensitizer that can competitively inhibit intrahepatic uridine nucleotides and the synthesis of mRNA and proteins, which further aggravates liver injury [14]. Therefore, the choice of LPS combined with D-GalN to induce acute liver injury in mice is a commonly used modeling method [15].

HT is found in forsythia, olive fruit, and olive tree branches [16], and exhibits promising biological efficacy in preventing obesity, diabetes, and neurodegenerative diseases. Furthermore, HT has anti-inflammatory, antitumor, and anti-thrombotic properties [17,18]. This study demonstrates the protective effects of HT against liver injury and inflammatory damage in a mouse model of LPS/D-GalN-induced acute lung injury (ALI). LPS/D-GalN-induced hepatotoxicity resulted in hepatocellular membrane destabilization and increased permeability, allowing the release of intracellular enzymes, such as ALT and AST [19], which are representative of the active substances, the activities of which are significantly increased in the bloodstream; thus, ALT and AST can be used as important indicators for judging the severity of liver injury [20,21]. In this study, we demonstrated that HT significantly reduced serum ALT, AST, and LDH activities and ameliorated pathological conditions such as cellular necrosis, endothelial hemorrhage, and inflammatory cell infiltration in the livers of mice with ALI induced by LPS/D-GalN.

The inflammatory response plays a crucial role in mediating the onset and progression of ALI, and inhibiting inflammation is an important strategy for the treatment of liver injury [22]. When the cell membrane is damaged and lysed, large amounts of content is released, thereby triggering an inflammatory response. IL-1β and TNF-α are important inflammatory factors involved in the inflammatory response, apoptosis, oxidative stress, and other processes, and have strong pro-inflammatory activities [23]. Leukocytes continuously exacerbate the inflammatory response under the stimulation of TNF-α, and phagocytes produce IL-6, which induces chemotaxis, cytotaxis, and bursting and exacerbation of pro-inflammatory factor expression [24]. In this study, the TNF-α and IL-6 levels in the liver tissue of mice in the M group were significantly increased. HT markedly inhibited the production of inflammatory factors, thereby alleviating the inflammatory response.

**Figure 7:**
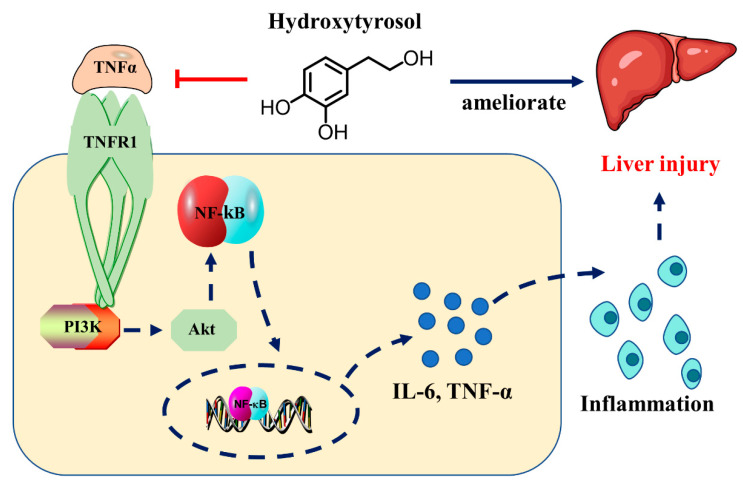
A schematic diagram showing HT mode of action.

These results suggest that, by modulating specific molecular pathways, HT has potential therapeutic effects for acute liver injury, providing a scientific basis for the further development of treatment strategies based on natural compounds. PI3K plays a role in TNF-α-induced cell damage and the activation of inflammatory pathways, and the PI3K/AKT signaling pathway is involved in various physiological and pathological cell processes, including proliferation and growth. The PTEN-mediated AKT/β-catenin signaling pathway enhances the proliferation and expansion of Lgr5+ hepatocytes [25], plays an important role in various liver diseases, such as alcoholic liver damage and pharmacological liver injury, and can cause tumors when disordered [26]. By inhibiting TNF-α signaling, HT subsequently inhibits the activation of the PI3K/AKT pathway. Whether it triggers the TNF alpha-AKT/beta-catenin pathway will be the focus of the next study [27,28]. NF-κB is one of the downstream substrates of AKT [29], and p65 is a subunit of NF-κB that activates the transcription process by directly binding to transcription elements, thereby initiating the transcription of a series of inflammatory factors and increasing their expression [30], such as TNF-α, IL-6, and ICAM-1. This thereby mediates hepatocyte inflammation and further triggers or exacerbates liver injury [31]. In this study, HT reduced the expression levels of TNF-α, IL-6, and VCAM-1, and attenuated liver injury (Figure 7).

In conclusion, this study demonstrated the ability of HT to attenuate the inflammatory response in mice with acute liver injury and revealed that it works by improving the TNF-α/PI3K/AKT signaling pathway. In future studies, we will explore the effect of HT on liver fibrosis to evaluate whether it can be used both as a long-term drug and as a backup drug for the clinical treatment of liver injury.

## 4. Materials and Methods

### 4.1. Plant Materials and Reagents

HT (hydroxytyrosol) (CAS: 10597-60-1, purity ≥ 98%) was purchased from Chengdu Alfa Biotechnology Co., Ltd. (Chengdu, China), and the LPS was purchased from Sigma (St. Louis, MO, USA). The positive control drug was SHUIFEI JIBIN JIAONANG (Tianjin Tasly Sants Pharmaceutical Co., Ltd.) (Tianjin, China). D-GalN was purchased from Shanghai Aladdin Biochemical Technology Co., Ltd. (Shanghai, China).

### 4.2. Animals

All the experiments and procedures performed in this study were approved by the Experimental Animal Ethics Committee of Henan University of Chinese Medicine (Ethical Review Approval No. IACUC-202307031, 16 June 2023–16 June 2028). Male Balb/C mice (weighing 18 ± 2 g, *n* = 50) were obtained from Zhejiang Vital River Laboratory Animal Technology Co., Ltd. (SCXK2024-0001, Pinghu City, China), and were housed in specific-pathogen-free (SPF) -grade animal laboratories at 18 °C–22 °C (Henan University of Chinese Medicine Animal Laboratory Center), alternating day and night, eating and drinking freely.

For this study, the mice were randomly divided into five groups (*n* = 10 for each group): the control group (CON), model group (M), positive group (Y, SHUIFEI JIBIN JIAONANG), low-dose HT + M group (HT-L, hydroxytyrosol-L), and the high-dose HT + M group (HT-H, hydroxytyrosol-H). In the low- and high-dose HT groups, HT was administered via gavage at a dose of 20, 40 mg/kg per day, and in the Y group, Y was administered via gavage at a dose of 48 mg/kg per day. The CON and M groups were given equal amounts of saline in the same manner. Gavage administration was performed once daily for 7 consecutive days. After 1 h of gavage on day 7, all mice received intraperitoneal injections of 700 mg/kg D-GalN + 10 μg/kg LPS for modeling, except for the mice in the CON group, which received saline. Blood was collected 6 h after modeling, centrifuged at 3000 rpm for 10 min at 4 °C, and divided and stored at −80 °C, and liver samples were stored at −80 °C for backup.

### 4.3. Pathology Assay

Liver tissues were fixed in 4% paraformaldehyde for 24 h, dehydrated through a graded series of alcohols, cleared with xylene, and embedded in paraffin wax. Five-micrometer sections were cut and stained with hematoxylin and eosin (H&E). Hematoxylin stain is a basic dye that causes the chromatin in the nucleus and the ribosomes in the cytoplasm to turn a purple-blue color; and eosin is an acidic dye that causes the components in the cytoplasm and extracellular matrix to turn a red color. Thus, morphological changes in the liver can be observed via HE staining.

### 4.4. Detection of Liver Biochemical Indicators in Serum

The level of ALT, AST, and LDH in the liver was measured using a commercial kit (C009-2-1, C010-2-1, A020-2, Nanjing Jiancheng Bioengineering Institute, Nanjing, China) following the manufacturer’s protocol. When the liver is inflamed, aminotransferases are released from the liver cells into the bloodstream. When the liver is diseased, serum aminotransferases are elevated, and their levels more than double when there is inflammation in one thousandth of the liver cells. Therefore, the amount of serum transaminase is an important indicator of the degree of liver disease.

### 4.5. RNA-Seq

The core of RNA-seq is the significance analysis of gene expression differences. Statistical methods were used to compare the gene expression differences between the CON, M, and HT groups, from which specific genes associated with the conditions were identified, and their biological significance was analyzed. Total RNA was extracted from liver tissue with TRIzol^®^ reagent (Invitrogen, Carlsbad, CA, USA). The purity and amount of RNA were assessed with TBS380 Picogreen (Invitrogen, Carlsbad, CA, USA). PCR fragments were identified using Certified Low Range Ultra Agarose (Bio-Rad, Hercules, CA, USA). Clusters were clustered using the HiSeq 4000 PE Cluster Kit (Invitrogen, Carlsbad, CA, USA) for subsequent sequencing, and finally, sequencing was performed using the HiSeq 4000 SBS Kit (300 cycles). Pathway enrichment analysis was conducted using the KEGG database to identify the pathways in which the genes were involved.

### 4.6. qRT-PCR Analysis

Fluorescence quantitative PCR was used to monitor the reaction process online in real time by labeling and tracking the PCR products with SYBR Green. The products were analyzed in combination with QuantStudio software 1.3.1 to calculate the initial concentration of the sample template to be tested to detect the mRNA gene expression levels. Total RNA was extracted from the liver tissue using a total RNA extraction kit (R1200, Solarbio, Beijing, China), and the RNA concentration was measured using a Nano Drop™ One ultraviolet-spectrophotometer (Thermo Scientific, Waltham, MA, USA). cDNA synthesis was performed using the SweScript RT I First Strand cDNA Synthesis Kit (G3330, Servicebio, Wuhan, China), and quantitative real-time PCR (qRT-PCR) was then performed using the 2× Universal Blue SYBR Green qPCR Master Mix (G3326-15, Servicebio, Wuhan, China) with the primers listed in Table 1.

### 4.7. Immunofluorescence Staining

Immunohistochemistry is based on the principle of the specific binding of an antigen to an antibody. The antigens in the tissue cells are identified through chemical reactions that cause color development in the chromogen that labels the antibody, and they are studied for localization, characterization, and quantification. Sequentially, the sections were put into xylene for 15 min, anhydrous ethanol for 5 min, 85% alcohol for 5 min, and 75% alcohol for 5 min, and they were then washed with distilled water. The sections were immersed in citrate buffer (PH 6.0), repaired in a microwave oven for 20min, and, after cooling, were washed with PBS three times for 5 min each time. The sections were incubated in 3% hydrogen peroxide at room temperature and protected from light for 25 min, and the slides were placed in PBS (PH 7.4) on a decolorizing shaker, shaken, and washed three times for 5 min each time. Bovine serum albumin was maintained at room temperature for more than 30 min, the sealing solution was gently shaken off, and the prepared primary antibody (CD31, VCAM1) was added dropwise via PBS according to a certain proportion on the section. The section was incubated flatly in a wet box at 4 °C overnight, and it was then washed with PBS three times for 5 min each time. The HRP-labeled secondary antibody was added dropwise and incubated at room temperature for 50 min, and then the section was washed with PBS three times for 5 min each time. FITC-tyramide was added to the section, which was incubated at room temperature for 10 min and then washed with PBS three times for 5 min each time. The images were captured using a fluorescence microscope, and their optical densities were determined using Image-Pro Plus 6.0.

### 4.8. Western Blot Analysis

Western blot (WB) was used to detect the expression of specific proteins within a sample via SDS-PAGE electrophoresis-transfer, primary and secondary antibody incubation, and color development. The total protein was extracted from the liver tissues using a whole protein extraction kit and was quantified using a bicinchoninic acid (BCA) protein assay kit. Sixty micrograms of protein from each sample was separated via sodium dodecyl sulfate-polyacrylamide gel electrophoresis (SDS-PAGE) and transferred to a nitrocellulose membrane. The membrane was blocked with bovine serum albumin (BSA) and incubated with primary antibodies targeting TNF-α, IL-6, PI3K, phosphorylated PI3K (p-PI3K), Akt, phosphorylated Akt (p-Akt), and β-actin. After washing with phosphate-buffered saline containing Tween 20 (PBST), a secondary antibody was added and incubated at room temperature. Following additional washes with PBST and phosphate-buffered saline (PBS), the protein levels were quantified using ImageJ2 software [32].

### 4.9. Cell Culture

The AML12 cell line, purchased from the Shanghai Cell Bank of the Chinese Academy of Sciences, was cultured in DMEM/F12 containing 10% FBS, 0.5% ITS-G and Dexamethasone and grown in a CO_2_ incubator with 5% CO_2_, 37 °C and saturated humidity. The logarithmic-growth-phase cells were taken to passage and then used for the experimentation. TNF-α gene overexpression (mouse: NM_013693) was induced using a PEI transfection reagent (HY-K2014, MCE).

AML12 cells in the logarithmic growth phase were divided into normal (CON), model (M) (10.0 μg/mL LPS), and HT (10 μM + 10.0 μg/mL LPS) groups and HT empty plasmid (CON + NC, 0.2/3 μg), TNF-α (0.2/3 μg) gene overexpression plasmid groups (TNF-α + HT, 0.2/3 μg).

### 4.10. In-Cell Western

Cells were fixed with 4% paraformaldehyde for 20 min at room temperature, followed by permeabilization with 0.1% Triton X-100 for five washes of 5 min each. The cells were blocked with 5% bovine serum albumin (BSA) for 90 min and incubated with primary antibodies targeting TNF-α (1:500 dilution), IL-6 (1:200 dilution), NF-κB p65 (1:400 dilution), and β-actin (1:400 dilution) overnight at 4 °C. After washing the cells with 0.2% phosphate-buffered saline containing Tween 20 (PBST), they were incubated with a secondary antibody (goat anti-rabbit IgG) and washed again. Images were captured using a dual-color infrared laser imaging system [33].

### 4.11. Statistical Analysis

Data were analyzed using SPSS 26.0 software (IBM, NewYork, NY, USA). One-way analysis of variance (ANOVA) was used to determine the statistical significance between the experimental groups and their respective controls. Data were presented as means ± SD. Unless otherwise specified, all results were statistically analyzed using GraphPad Prism 8.0 software. Statistical significance was determined at *p* < 0.05 (*) and *p* < 0.01 (**).

## 5. Conclusions

Our findings suggest that HT has therapeutic potential in the treatment of acute liver injury (ALI) by mitigating inflammatory damage and TNF-α may be a key target for its beneficial effects.

## Figures and Tables

**Table 1:** Sequences of the primers for qRT-PCR.

Gene	Forward Primer	Reverse Primer
*VCAM-1*	GGAAAAGCTCTTGTTTGCCG	ATTGTCACAGCACCACCCTCTT
*CXCL5*	AGTGCCCTACGGTGGAAGTCATA	AGTGCATTCCGCTTAGCTTTCTT
*NLRP3*	TAAGAACTGTCATAGGGTCAAAACG	GTCTGGAAGAACAGGCAACATG
*TNF-α*	CCCTCACACTCACAAACCACC	CTTTGAGATCCATGCCGTTG
*GAPDH*	CCTCGTCCCGTAGACAAAATG	TGAGGTCAATGAAGGGGTCGT

## Data Availability

All data supporting the findings of this study are include within the article.
